# Prevalence analysis of OX40-positive cell populations in solid tumors

**DOI:** 10.1186/2051-1426-3-S2-P113

**Published:** 2015-11-04

**Authors:** James Ziai, Gretchen Frantz, Liping Zhang, Cleopatra Kozlowski, Houston Gilbert, Jacquelyn Smith, Marcin Kowanetz, Jeong M Kim, Mahrukh Huseni

**Affiliations:** 1 Genentech, Inc., South San Francisco, CA, USA; 2 Ventana Medical Systems, Inc., Tucson, AZ, USA

## Background

Increased numbers of OX40+ T cells within the primary tumor of colorectal cancer (CRC) patients has been associated with improved survival. Similarly, OX40 expression in sentinel lymph nodes is inversely associated with tumor grade and clinical stage in melanoma patients. However, the prevalence of OX40+ tumor-associated lymphocytes remains underreported in many human solid tumor types. In this study, we report prevalence of OX40 by chromogenic immunohistochemistry in melanoma, colorectal, bladder, renal cell (RCC), triple-negative breast (TNBC), non-small cell lung (NCSCLC) and ovarian cancers and its association with available clinical parameters.

## Methods

Tumor-associated OX40+ cells in melanoma (n=40), bladder (n=51), CRC (n=40), NSCLC (n=40), TNBC (n=45), ovarian (n=40) and RCC (n=42) patient samples including primary site and a subset of matched metastases have been stained for OX40 using a DAB-based chromogenic immunohistochemistry assay. Stained sections have been independently scored for OX40+ lymphocyte area percentage by two pathologists and a subset will be digitally imaged and analyzed. Image analysis includes OX40+ lymphocyte enumeration and area calculation within pathologist-defined regions of interest (ROI) to include the tumor and surrounding stroma. OX40 prevalence (OX40-positive cells/mm^2^ and % OX40+ cell area) were compared with patient age, clinical parameters, and outcome, where available, to determine potential associations.

## Results

Results to be reported include a.) pathologist scoring of OX40+ cell prevalence b.) image analysis of OX40+ cell number and area percentage in a subset of indications c.) concordance between image analysis and pathologists' scores d.) OX40 prevalence by tumor type e.) OX40 prevalence and association with clinical features, where available, and statistical significance.

## Conclusions

Prevalence of OX40 expression and association with clinical features are first reported in some solid tumor types.

